# The Oldest Hospital for Sick Children in the United Kingdom

**Published:** 1908-06-13

**Authors:** Stewart Johnson

**Affiliations:** Secretary. The Hospital for Sick Children, Great Ormond Street, London, W.C.


### THE HOSPITAL FOR SICK CHILDREN, Great Ormond Street, W.C.

### EDITOR'S LETTER-BOX.

**Our correspondents are reminded that prolixity is a great bar to publication, and that brevity of style and conciseness of statement greatly facilitate early insertion.**

### THE OLDEST HOSPITAL FOR SICK CHILDREN IN THE UNITED KINGDOM.

Sir,—To prevent misconception such as has occasionally arisen in the past, and which might arise again in the future, from the recent letter to the papers from the Secretary of the Royal Waterloo Hospital, may I ask you to state that the first hospital for sick children to be established in this country was the Hospital for Sick Children, Great Ormond Street. The treatment which the Royal Waterloo Hospital afforded before 1852, the year of the foundation of the Great Ormond Street Hospital, and for many years after that date was dispensary treatment only. How great was the need of a children's hospital at that time may be gathered from the fact that in January 1842, out of 2,363 persons in all the London hospitals only 26 were children under the age of 10; while of 50,000 persons dying annually in the metropolis 21,000 were children under that age.

Yours faithfully,
Stewart Johnson, Secretary.

The Hospital for Sick Children, Great Ormond Street, London, W.C., June 9.

